# Light at the end of the channel: optical manipulation of intrinsic neuronal excitability with chemical photoswitches

**DOI:** 10.3389/fnmol.2013.00005

**Published:** 2013-03-21

**Authors:** Alexandre Mourot, Ivan Tochitsky, Richard H. Kramer

**Affiliations:** ^1^Department of Molecular and Cell Biology, University of CaliforniaBerkeley, CA, USA; ^2^Department of Neurobiology of Adaptive Processes, UMR7102 CNRS, Université Pierre et Marie CurieParis, France

**Keywords:** optochemical genetics, optogenetics, chemical genetics, photopharmacology, photoswitches, ion channels, azobenzene

## Abstract

Ion channels are transmembrane proteins that control the movement of ions across the cell membrane. They are the molecular machines that make neurons excitable by enabling the initiation and propagation of action potentials (APs). Rapid signaling within and between neurons requires complex molecular processes that couple the sensing of membrane voltage or neurotransmitter release to the fast opening and closing of the ion channel gate. Malfunction of an ion channel's sensing or gating module can have disastrous pathological consequences. However, linking molecular changes to the modulation of neural circuits and ultimately to a physiological or pathological state is not a straightforward task. It requires precise and sophisticated methods of controlling the function of ion channels in their native environment. To address this issue we have developed new photochemical tools that enable the remote control of neuronal ion channels with light. Due to its optical nature, our approach permits the manipulation of the nervous system with high spatial, temporal and molecular precision that will help us understand the link between ion channel function and physiology. In addition, this strategy may also be used in the clinic for the direct treatment of some neuronal disorders.

## Introduction

Light is the ideal external stimulus for controlling biological processes with high accuracy: it can be manipulated with very high spatial and temporal precision, it can be projected onto a tissue from afar and, because cells are typically not intrinsically photosensitive, light can be used as an orthogonal, highly specific stimulus. In the last decade, research in neuroscience has resulted in an explosion of strategies aiming at optically controlling electrical signals, by rendering ion channels and neurons light-sensitive. These approaches include naturally photosensitive proteins and protein domains, caged neurotransmitters and reversible photoswitches (Kramer et al., 2009; Miesenbock, 2011; Brieke et al., 2012). The microbial opsins channelrhodospin, halorhodopsin and archaerhodopsin as well as other light-sensitive ion channels and transporters have revolutionized neuroscience, by enabling the remote control of neuronal excitability (Boyden et al., 2005; Zhang et al., 2007; Chow et al., 2010). The incredible success of these optogenetic tools lies in their versatility (they are one-component systems, as the chomophore retinal is naturally present in all vertebrate tissues) and their ability to be specifically targeted to desired neuronal populations (Fenno et al., 2011; Yizhar et al., 2011; Dugué et al., 2012). By overriding the electrical activity of neurons, microbial opsins can bring the membrane potential closer to or further away from the threshold for AP firing. These opsins can thus be used to link optically induced changes in activity of defined neurons with resultant circuit or behavioral effects. While advantageous for comprehending neural networks at a systems level, the optogenetic approach is ill-suited for understanding the intrinsic mechanisms regulating neuronal excitability on a molecular and cellular level. Light-sensitive modules from plants, such as phytochromes, cryptochromes, LOV domains or BLUF domains can be genetically coupled to mammalian proteins in order to gain optical control over protein–protein interactions, protein or DNA binding, enzymatic activity or subcellular protein localization (Möglich and Moffat, 2010; Pathak et al., 2012). LOV domains have been fused to the C-terminal fragment of Orai1 calcium channels to remotely control Ca^2+^ signaling (Pham et al., 2011). While attractive, this strategy may prove difficult to transpose to other ion channels. In contrast, caged compounds and reversible photoswitches are chemical molecules that have been used by neuroscientists to modulate the activity of endogenous ion channels in neurons (Goeldner and Givens, 2005). Cages allow for the photorelease of neurotransmitters and secondary messengers, triggering signaling events with temporal and spatial precision (Ellis-Davies, 2007; Warther et al., 2010). However, since uncaging is an irreversible photochemical process, the duration of the elicited effect is dependent on the kinetics of diffusion or re-uptake of the photoreleased molecule. On the other hand, chemical photoswitches (Gorostiza and Isacoff, 2008; Beharry and Woolley, 2011; Fehrentz et al., 2011) can be used to modulate the activity of ion channels in a reversible fashion, allowing for many rounds of activation/deactivation (or inhibition/disinhibition). In this review we will describe our recent work on developing both one- and two-component photoswitchable systems for the optical control of voltage-gated ion channels and neuronal excitability.

## One-component systems: development and tuning of photochromic ligands

**Figure 1:**
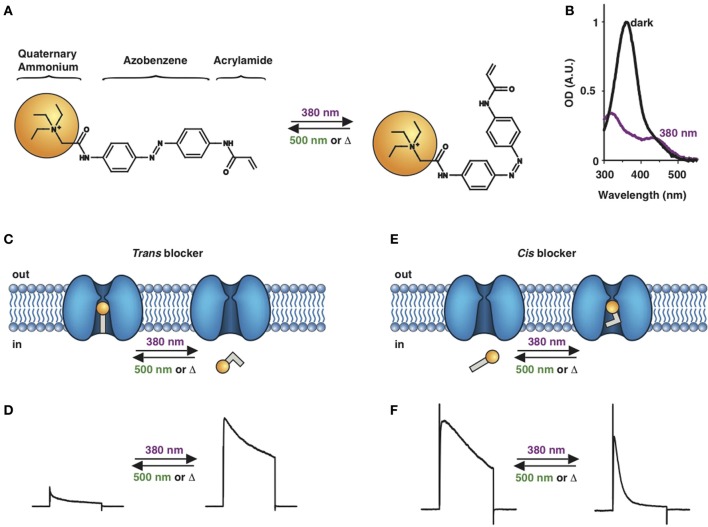
**Photoswitchable blockers for voltage-gated potassium channels. (A)** Chemical structures of *trans* (left) and *cis* (right) AAQ (Acrylamide-Azobenzene-Quaternary ammonium). The quaternary ammonium moiety (ligand) is highlighted by a yellow sphere. **(B)** Absorption spectrum of QAQ (Quaternary ammonium-Azobenzene-Quaternary ammonium) in the dark (~100% *trans*) and during illumination with 380 nm light (mostly *cis*). **(C)** Scheme of a *trans* blocker. The blocker binds to the intracellular vestibule and blocks ion conduction through voltage-gated potassium channels in the *trans* configuration (500 nm light or darkness) but not after photoisomerization to *cis* (380 nm). **(D)** Example of a voltage-clamp recording of current through voltage-gated potassium channels after depolarization of the membrane, using a *trans* blocker under 500 or 380 nm light illumination. **(E)** Scheme of a *cis* blocker. The blocker binds to the intracellular vestibule and blocks ion conduction through voltage-gated potassium channels in the *cis* configuration (380 nm light) but not in the dark or after photoisomerization to *trans* (500 nm). **(F)** Example of a voltage-clamp recording of current through voltage-gated potassium channels after depolarization of the membrane, using a *cis* blocker under 500 or 380 nm light illumination. Δ: thermal relaxation in the dark.

Photochromic ligands (PCLs) are light-sensitive molecules that can photo-isomerize after absorption of a photon. Photoisomerization induces a modification of the spectral, chemical, electronic and steric properties of the ligand, in a manner that can affect its biological activity (Beharry and Woolley, 2011; Fehrentz et al., 2011). Several properties of azobenzene have made it the most-commonly used photoswitch for biological applications: (1) the *trans* to *cis* photoisomerization results in a drastic change in geometry (from planar to twisted, Figure 1A) and polarity (from ~0 to 3 Debye) (2) the absorption spectra of the two isomers are different enough (Figure 1B) to allow nearly-full conversion to *cis* or *trans* under the appropriate light conditions; (3) azobenzenes are relatively small, simple chemical moieties that can easily be conjugated to various ligands; (4) photoisomerization occurs on a picosecond time scale, orders of magnitude faster than most biological processes, and (5) azobenzenes are very photostable, allowing many cycles of photoisomerization without fatigue. Because the *trans* isomer is thermodynamically more stable than the *cis* isomer, it is the highly predominant form in the dark (>99.99%).

Photoregulation of ion channels using PCLs was pioneered with the nicotinic acetylcholine receptor (nAChR) in the late 1960s (Deal et al., 1969; Bartels et al., 1971; Lester et al., 1979; Krouse et al., 1985) but only recently adapted to other ion channels, such as the ionotropic glutamate (Volgraf et al., 2007; Stawski et al., 2012) and gamma-amino butyric acid receptors (GABARs) (Stein et al., 2012; Yue et al., 2012). Following a similar strategy, we have developed a small library of PCLs that block voltage-gated potassium channels (K_v_s) in a photo-reversible fashion (Fortin et al., 2008; Banghart et al., 2009; Mourot et al., 2011, 2012; Fehrentz et al., 2012). Quaternary ammoniums (QAs) inhibit K_v_s by entering the intracellular vestibule and blocking K^+^ conduction (Choi et al., 1993). We designed photoswitchable K_v_ blockers with a central azobenzene core, flanked on one side by a QA “head” and on the other side by a hydrophobic “tail”. The initial “tail” was an acrylamide moiety, and the compound was named Acrylamide-Azobenzene-Quaternary ammonium (AAQ) (Figure 1A) (Fortin et al., 2008). The reactive acrylamide group was chosen because AAQ was originally designed to covalently attach to the outer mouth of native K_v_s (Fortin et al., 2008), but further investigation revealed that, due to its hydrophobic “tail,” AAQ was in fact able to cross the cell membrane and non-covalently bind to the internal vestibule (Banghart et al., 2009). AAQ is a *trans* blocker of K_v_s, that is, the *trans* isomer has a much higher affinity for the internal vestibule than the *cis* form. In the dark or under 500 nm illumination, channels will be blocked by AAQ, whereas 380 nm light will relieve the blockade and restore K^+^ conduction (Figures 1C,D). AAQ is a *trans* blocker for the Shaker K^+^ channel, but also for a wide range of mammalian K_v_s heterologously expressed or naturally present in neurons (Table 1).

**Table 1:** **Photochemical and pharmacological properties of a series of photoswitchable ion channel blockers**.

**PCL**	**Chemical structure**	**Ion channels tested**			**λ_max_ (nm)**	**t_1/2_**	**Conc. (μM)**	**References**
		***Trans* block**	***Cis* block**	**No block**				
AAQ	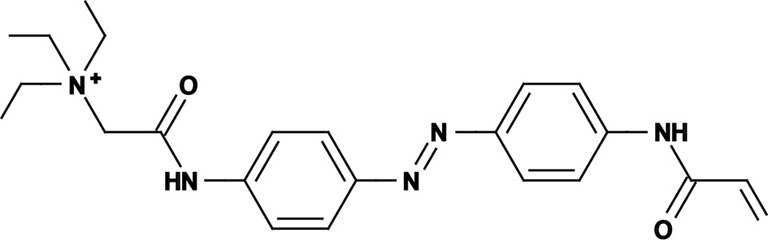	Shaker^a^		K_v_3.1^a^	364	11 min^f^^,^ ^j^	150–1000	Fortin et al., 2008; Banghart, 2009; Banghart et al., 2009; Polosukhina et al., 2012
		K_v_1.2^a^		Na_v_1.2^a^				
		K_v_1.3^a^		L-type Ca_v_^c^				
		K_v_1.4^a^					
		K_v_2.1^a^						
		K_v_3.3^a^						
		K_v_4.2^a^						
		K_v_^b^^,^ ^e^						
BzAQ	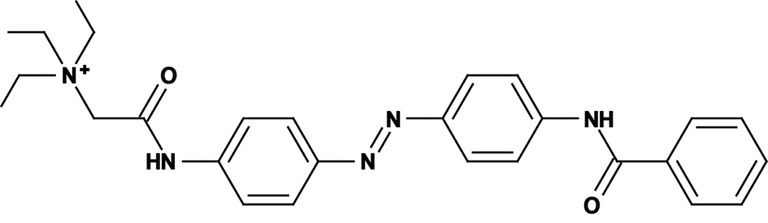	Shaker^a^		Na_v_^b^^,^ ^c^	367	4 min^f^^,^ ^j^	20–30	Banghart, 2009; Banghart et al., 2009
		K_v_^b^		L-type Ca_v_^c^				
PrAQ	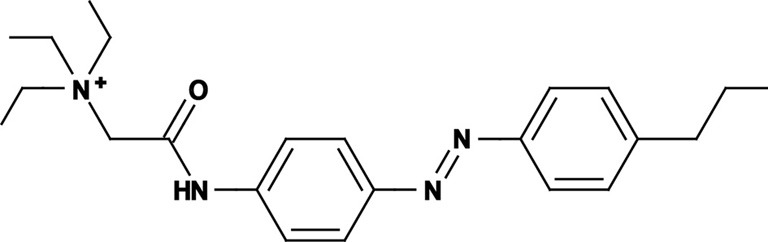		Shaker^a^		338	13 min^f^^,^ ^j^	40	Banghart, 2009; Banghart et al., 2009
DENAQ	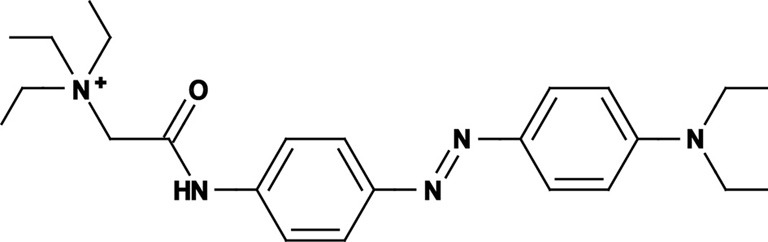	K_v_3.1^a^		Shaker^a^	470	300 ms^k^	100	Mourot et al., 2011
		K_v_2.1^a^^,^ ^f^		K_ir_2.1^a^^,^ ^f^				
		K_v_4.2^a^^,^ ^f^		Ca_v_2.2^a^^,^ ^f^				
		K_v_^b^^,^ ^f^		Na_v_^f^				
PhENAQ	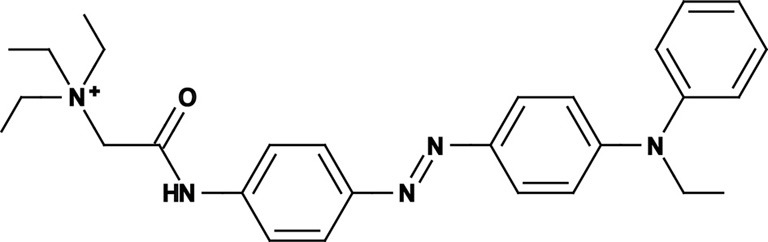		Shaker^a^		456	160 ms to 2.6 s^k^	20–50	Mourot et al., 2011
			K_v_3.1^a^^,^ ^f^					
			K_v_^b^						
QAQ	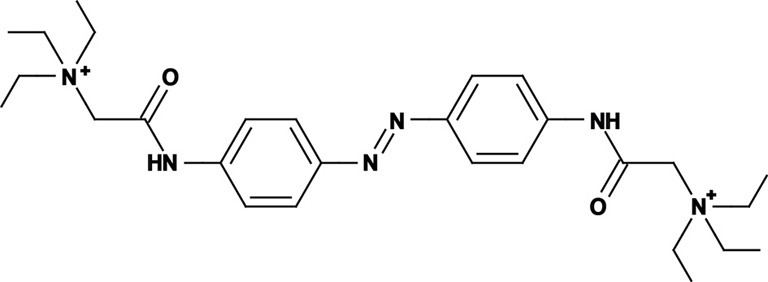	Shaker^a^		K_ir_2.1^a^	362	7 min^j^	100–300^l^	Banghart et al., 2009; Mourot et al., 2012
		K_v_2.1^a^		HCN^g^				
		K_v_3.1^a^		iGluR^b^				
		K_v_4.2^a^						
		K_v_^b^^,^ ^i^						
		Na_v_^b^^,^ ^g^^,^ ^h^^,^ ^i^						
		Na_v_1.5^a^^,^ ^f^						
		Ca_v_2.2^a^						
		L- type Ca_v_^c^						
2,2′-dimethoxy-QAQ	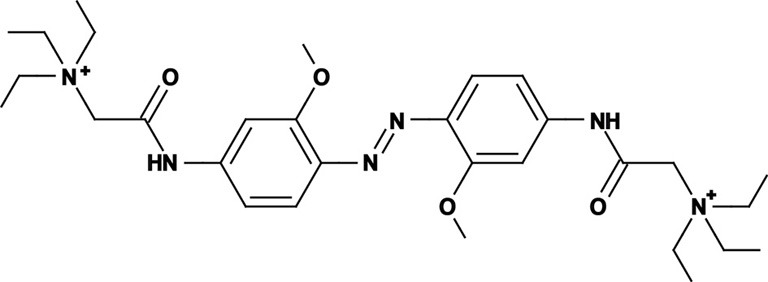	Shaker^a^			394	nd^m^	350–1000^l^	Fehrentz, 2012; Fehrentz et al., 2012
		Na_v_^d^						
2,6-dimethyl-QAQ	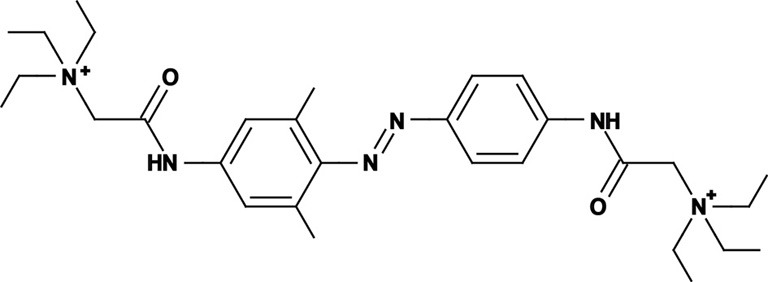	Shaker^a^			340	39 h^f^^,^ ^j^	50–100^l^	Fehrentz, 2012; Fehrentz et al., 2012
		Na_v_^d^						
2,2′,6,6′-tetramethyl-QAQ	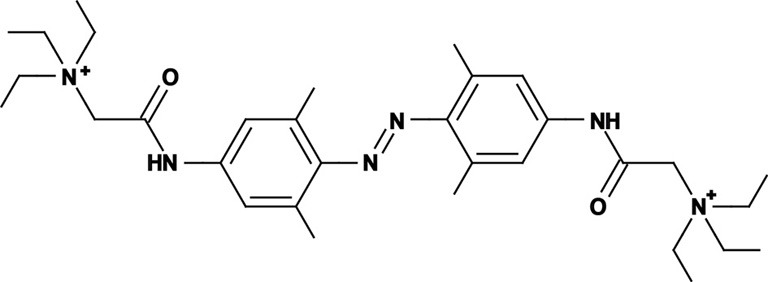	Shaker^a^			333	4.5 d^j^^,^ ^n^	100^l^	Banghart, 2009; Fehrentz, 2012; Fehrentz et al., 2012
		Na_v_^d^						

The table indicates the chemical structures and values for the maximal absorption in aqueous solution (λ_max_) for the trans isomer, the values for thermal relaxation of the cis isomer in the dark (t_1/2_) as well as the working concentrations typically used in our studies (Conc.).

a Heterologous expression system.

b Dissociated rat hippocampal neurons.

c GH3 cells.

d NG108-15 cells.

e Retinal ganglion cell.

f Unpublished results.

g Acutely dissociated trigeminal neurons.

h TTX-resistant Na_v_ from acutely dissociated trigeminal neurons.

i Second order neurons in mouse spinal cord slices.

j Thermal relaxation rate measured in phosphate buffer pH7.4 using a spectrophotometer.

k Thermal relaxation rate estimated from electrophysiological recordings.

l Compound included in the patch pipette.

m Thermal relaxation too fast to be measured in aqueous solution.

n Thermal relaxation not determined for 2,2′,6,6′tetramethyl-QAQ but for a compound very similar, having the same tetramethyl azobenzene core, but tri-methyl ammoniums groups instead of tri-ethyl ammonium groups.

nd: not determined.

Each biological application requires a specific set of characteristics. Photoswitchable K_v_ blockers can be easily modified using the power of organic chemistry, enabling us to generate a small library of compounds with various pharmacological and photophysical properties (Table 1) (Banghart, 2009; Mourot et al., 2013). AAQ is a permanently charged molecule that must cross the cell membrane to photosensitize K^+^ channels. Membrane permeation has been experimentally verified for AAQ (Banghart et al., 2009) and has been observed for other permanently charged drugs like the local anesthetics tonicaine (Wang et al., 1995) and tetra-pentylammonium (Taglialatela et al., 1991). In all the aforementioned cases, the charged molecule accumulates inside the cell where it is trapped for hours, more than 24 h in the case of AAQ loaded into dissociated hippocampal neurons (Banghart et al., 2009). Replacing the acrylamide “tail” with a more hydrophobic benzylamide moiety results in a molecule (BzAQ) with better membrane permeation, as evidenced by the drastic increase in potency when the PCLs are applied outside cells (see working concentration for AAQ an BzAQ in Table 1) but a similar potency when the compounds have direct access to the internal vestibule (Banghart et al., 2009). Doing the opposite, i.e., replacing the acrylamide chain with a more polar, charged QA group results in a compound (QAQ) ineffective at crossing the cell membrane. Of note, some of the most hydrophobic compounds, e.g., PhENAQ, suffer from lack of water solubility.

Chemical modifications can also affect the pharmacology of the PCLs. AAQ photosensitizes most, but not all K_v_s; for example, current through K_v_3.1 is not affected by AAQ (Fortin et al., 2008). Similarly, DENAQ photosensitizes many K_v_s but not Shaker which belongs to the K_v_1 family (Mourot et al., 2011). QAQ, on the other hand, not only photosensitizes K_v_s but also voltage-gated Na^+^ and Ca^2+^ channels (Na_v_s and Ca_v_s, respectively) (Mourot et al., 2012), which are structurally related to K_v_s and also blocked by intracellular QAs (Scholz, 2002). Of all the PCLs tested, only the ones with two QA groups seem to block Na_v_s (Table 1). The identity of the biologically active isomer also depends on the chemical nature of the “tail.” For example AAQ, BzAQ, and QAQ are *trans* blockers of K_v_s, that is, they block preferentially in the *trans* configuration, but other PCLs such as PrAQ or PhENAQ are *cis* blockers (Table 1 and Figures 1E,F). In the absence of illumination, PCLs exist in only one form: the thermally stable *trans* isomer. *Cis* blockers can be advantageous for certain applications as they are inert in the dark (assuming the *trans* isomer does not block at all) and activate only after illumination with the appropriate wavelength of light. Understanding the molecular features necessary for the recognition of the *cis* or the *trans* isomer by various ion channels is crucial to the rational design of more selective PCLs. Alas, this is not a straightforward task, since it requires the synthesis and screening of a large library of compounds in addition to molecular modeling of the PCLs bound to the channel.

The wavelength used to isomerize AAQ from *trans* to *cis* is in the near-UV range (380 nm), which is compatible with biological systems. However, there is a clear interest in developing PCLs with a red-shifted spectrum, as longer wavelengths of light are less phototoxic and penetrate deeper into tissue. Many of the phenyl ring substituents are known to alter the spectral characteristics of azobenzenes and this effect can be rationally utilized to design novel azobenzene derivatives (Rau, 1973; Beharry and Woolley, 2011). For example, electron donating groups at the *ortho* or *para* positions can red-shift the absorption spectrum of the *trans* isomer considerably (Chi et al., 2006; Sadovski et al., 2009; Beharry et al., 2011, 2008; Stawski et al., 2012). We have designed PCLs where the amide tail of AAQ was replaced by electron donating amine tails (see DENAQ and PhENAQ in Table 1). The new PCLs are red-shifted by up to 100 nm, enabling the photocontrol of K_v_s with blue light (480 nm) (Mourot et al., 2011). The Trauner group has also developed derivatives of QAQ with electron donating groups in the *ortho* position (see 2,2′-dimethoxy QAQ in Table 1), but this resulted in only a minor red-shift (~30 nm) and in some cases compounds that were ineffective at blocking voltage-gated ion channels (Fehrentz, 2012; Fehrentz et al., 2012). The internal vestibule of K_v_s may be too tight to accommodate bulky substituents in the *ortho* position, but seems to accommodate rather long, bulky, hydrophilic or hydrophobic “tails” in the *para* position.

Once light is turned off, the *cis* isomer relaxes back to the more stable *trans* form, yielding essentially 100% of the *trans* isomer. The kinetics of thermal relaxation are highly dependent on the substituents located on the phenyl rings of the azobenzene (Beharry and Woolley, 2011). For example, the half-life of *cis* QAQ in aqueous solution (t_1/2_) is in the 7 min range, but can be extended to hours and even days after adding methyl groups in the *ortho* position (see di- and tetra-methyl QAQs in Table 1). Continuous light exposure is not required to maintain these thermo-stable compounds in their *cis* form. This can be advantageous in cases where constant illumination would be toxic, impractical or simply not technically feasible. Conversely, thermal relaxation can also be sped up. The fastest thermal relaxation reported to date for an azobenzene derivative in water is in the nanosecond range (Garcia-Amorós et al., 2012). Rapid thermal relaxation is desirable, since a single wavelength of light can then be used to quickly toggle the photoswitch between its two isomers. However, it is not necessarily ideal, since very intense illumination is required to accumulate enough of a very short-lived isomer to produce a significant biological effect. The red-shifted PCLs we have engineered have much faster relaxation kinetics than QAQ, too fast, in fact, to be measured using classical spectrophotometers (Fehrentz et al., 2012), but estimated to be in the hundreds of millisecond range using electrophysiological recordings (Mourot et al., 2011). This value is in agreement with the reported half-lives in the tens of milliseconds range for similar amino-azobenzenes (Chi et al., 2006). Despite their short half-lives, some red-shifted PCLs block a considerable fraction of the channel's ionic current, suggesting a significant accumulation of the *cis* isomer (Mourot et al., 2011; Fehrentz et al., 2012). Importantly, because the spectra of the two amino-azobenzene isomers overlap substantially (Beharry and Woolley, 2011), light cannot be used to trigger the reverse isomerization to the *trans* form. Therefore, rapid thermal relaxation is a necessary feature for the rapid and reversible photocontrol of neuronal activity with such red-shifted azobenzenes. New types of red-shifted azobenzenes show a substantial separation in the spectra of the two isomers, making it possible to use two different wavelengths of visible light for rapidly accumulating a high percentage of either the *cis* or the *trans* isomer (Beharry et al., 2011; Bléger et al., 2012).

What fraction of the *cis* isomer can be generated under our experimental conditions? Since *cis* and *trans* azobenzenes have partially overlapping spectra, illumination produces a photostationary state with a certain percentage of each isomer. Using the appropriate wavelength of light, a large fraction of the photoswitch molecules can be isomerized to *cis* or to *trans* (Beharry and Woolley, 2011). Precise determination using ^1^H NMR revealed that up to 95% of the photoswitch can be converted to *cis* under illumination with 380 nm (Gorostiza et al., 2007; Banghart and Trauner, 2013). In order to obtain an estimate of the percentage of the *cis* isomer under physiological conditions, we measured the concentration of two PCLs, AAQ and BzAQ, that blocked half of the Shaker K^+^ channel current, both in the dark and under 380 nm illumination (IC_50(dark)_ and IC_50(380 nm)_, respectively) (Banghart et al., 2009). These two PCLs have identical azobenzene chromophores, with two amide groups in the *para* position, and therefore should have similar photochemical properties. We found the IC_50(380 nm)_/IC_50(dark)_ ratio to be 28 and 42 for AAQ and BzAQ, respectively. Assuming *cis* AAQ and *cis* BzAQ bind to Shaker K^+^ channels with negligible affinity, the percentage of the *trans* molecule under 380 nm light illumination is 1/28 = 3.5% for AAQ, and 1/42 = 2.4% for BzAQ. If the affinity of *cis* AAQ or *cis* BzAQ for Shaker K^+^ channels is not negligible, then the percentage of the *trans* PCL under 380 nm light is even lower. Hence, it is possible to obtain more than 96% photoisomerization to the *cis* form using AAQ, BzAQ or other PCLs with similar photoswitches. As mentioned above, the situation may be different with quickly relaxing compounds like DENAQ or PhENAQ.

## Optical control of action potential firing with PCLs

The molecular mechanism underlying the initiation and propagation of AP is the opening and closing of voltage-gated ion channels (Hille, 2001). When excitatory inputs to a neuron trigger a depolarization above the AP initiation threshold (typically −50 mV), Na_v_s quickly open and sodium ions rush into the cell, depolarizing the membrane even further. After a slight delay, Na_v_s inactivate and K_v_s start to open, thus generating a potassium efflux that repolarizes the cell membrane. K_v_s and other K^+^ channels (e.g., calcium-activated K^+^ channels) do not close immediately after the membrane returns to its resting membrane voltage, generating a hyperpolarization phase called afterhyperpolarization (AHP), during which the neuron is refractive to subsequent AP. Therefore, the influx and efflux of sodium and potassium ions shape APs, and rapidly photoregulating their flux should give us control over AP firing.

**Figure 2:**
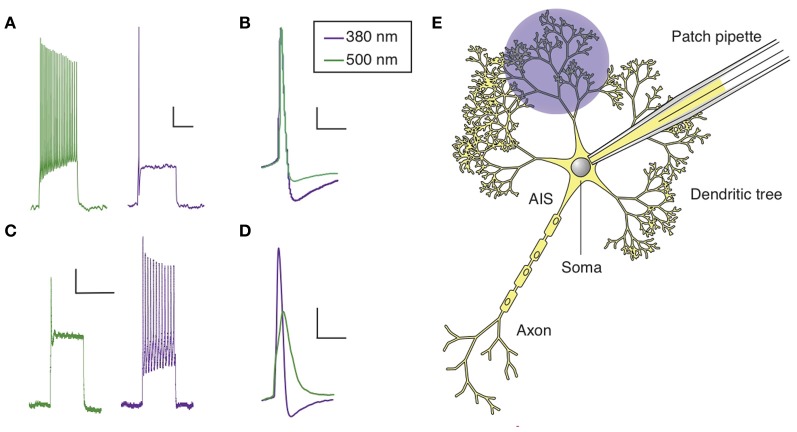
**Optical control of AP firing in hippocampal neurons using PCLs. (A)** Current clamp recording of a hippocampal neuron in culture treated with AAQ, showing the effect of light on neuronal excitability. **(B)** AP shape in both wavelength of light for an AAQ-treated hippocampal neuron. **(C)** Current clamp recording of a hippocampal neuron in culture loaded with QAQ through the patch pipette, showing the effect of light on spike inhibition. **(D)** AP shape in both wavelength of light for a neuron loaded with QAQ. **(E)** Patterned illumination for the optical control of the excitability of a dendritic tree using QAQ loaded through the patch pipette. The main regions of a neuron are indicated. Scale bars **(A,C)** 10 mV, 500 ms; **(B,D)** 10 mV, 25 ms.

When neurons are at rest, some K_v_s, notably the low-voltage activated ones, are open and play a role in maintaining and regulating the membrane potential (Hille, 2001). Blocking them can result in membrane depolarization and increase neuronal activity. AAQ, which is a photoswitchable blocker selective for K^+^ channels, can be used to optically increase neuronal excitability and induce AP firing (Fortin et al., 2008). In the dark or under green light, AAQ blocks neuronal K_v_s, depolarizes the cell membrane and enables high-frequency firing (Figure 2A). Subsequent photoisomerization to *cis* with 380 nm light, which unblocks K_v_s, hyperpolarizes the membrane and reduces neuronal excitability. Comparing the AP shapes under both wavelengths of light clearly shows that the peak is unaltered, while hyperpolarization is reduced under 500 nm light, consistent with the selective block of K^+^ channels (Figure 2B). Optical regulation of membrane excitability with AAQ works well not only in cultured neurons, but also in rat cerebellar slices, in the heart central pattern generator of leeches, as well as in mice retinal neurons *in vitro* and *in vivo* (Fortin et al., 2008; Polosukhina et al., 2012). One limitation of AAQ is that, in the absence of illumination, the *trans* configuration dominates, leading to a tonic block of K_v_s and potential cytotoxicity. *Cis* blockers like PhENAQ could offer significant advantage by minimizing basal excitation in the dark (Mourot et al., 2011).

QAQ has a different effect on neuronal excitability: it does not modulate the resting membrane potential, but prevents AP firing when neurons receive excitatory inputs, by blocking all voltage-gated ion channels, i.e., K_v_s, Na_v_s and Ca_v_s (Mourot et al., 2012). When illuminated with 500 nm light, neurons loaded with QAQ fire a single AP at the onset of current injection, followed by depolarization block and absence of repetitive spiking (Figure 2C). The presence of the first spike can be attributed to the nature of the molecular interaction between *trans* QAQ and ion channels: QAQ is an open-channel blocker, that is, ion channels must first open before QAQ can bind and block ion conduction (Hille, 2001; Mourot et al., 2012). Under 380 nm light, ion-channel block is relieved and neurons can fire at a high-frequency. Due to the block of both K_v_s and Na_v_s, the AP amplitude is reduced and its half-width increased under 500 nm compared to 380 nm light (Figure 2D). QAQ blocks Na_v_s only partially, yet it potently silences cells, probably because its blockade of K^+^ channels potentiates blockade of Na^+^ channels (Drachman and Strichartz, 1991).

Neuronal dendrites receive, coordinate and integrate thousands of excitatory and inhibitory inputs (Magee and Johnston, 2005). Inputs are integrated at the level of the soma and APs are initiated in a specialized, unmyelinated element of the neuron called the axon initial segment (AIS), before they propagate through the rest of the axon (Kole and Stuart, 2012). In some neurons, such as hippocampal and cortical pyramidal neurons, APs can also back-propagate from the soma to the dendrites, which is essential to many forms of synaptic plasticity (Magee, 1997; Markram, 1997; Magee and Johnston, 2005). The presence of a high density of specialized voltage-gated ion channels is essential to the electrical properties of AIS, axons and dendrites. Yet, manipulating these ion channels in specific sub-cellular domains of a neuron is technically challenging. Local perfusion of tetrodotoxin, a specific Na_v_ blocker, has been used to show that back-propagation of APs is required for the induction of synaptic plasticity (Magee, 1997), but this can only be done in large neurons with dendritic trees long-enough to confine tetrodotoxin to the desired location. Membrane-impermeant ion-channel blockers like QX-314 can also be used to eliminate excitability in dendrites and axons of single neurons, making it possible to pharmacologically isolate synaptic from non-synaptic events and elucidate the composition of the synaptic responses (Smirnov et al., 1999; Wilent and Contreras, 2005). Because QX-314 is membrane-impermeant (Strichartz, 1973; Binshtok et al., 2007), it can be dialyzed into cells through a patch pipette, but once inside, it cannot be removed; therefore its action is irreversible. In contrast to chemical ligands, light can be delivered onto tissues with great spatial resolution, and recent optical techniques even allow for patterned illumination in three dimensions (Vaziri and Emiliani, 2012). Therefore, photoconvertible ligands such as the ones we have developed could be extremely useful in understanding the roles of voltage-gated ion channels in controlling dendritic and axonal excitability, especially in compact neurons. QAQ, which is essentially a photoswitchable derivative of QX-314 (Mourot et al., 2012), can open the way to new experimental opportunities. Because light can be projected with great spatial precision, it might be possible to control APs in particular parts of a neuron, such as a branch of the dendritic tree or the AIS (Figure 2E). For example, QAQ could be used to reversibly block back-propagating APs with a cellular precision that is hard to achieve with externally applied blockers (Magee, 1997), and help evaluate the contribution of dendritic excitability to synaptic integration and plasticity.

## Cell targeting with QAQ

Optogenetic tools have one advantage over PCLs: they can be targeted to genetically defined cell types using either specific promoters or cre recombinase-inducible expression systems in conjunction with transgenic animals expressing cre in specific population of neurons (Fenno et al., 2011; Yizhar et al., 2011; Dugué et al., 2012). Cell targeting is essential for establishing a causal relationship between the activity pattern of a defined population of neurons and the function of that neuronal population at the circuit or behavioral level. Even though QAQ is not genetically encodable, we have designed a strategy for delivering it to a defined sub-population of neurons.

**Figure 3:**
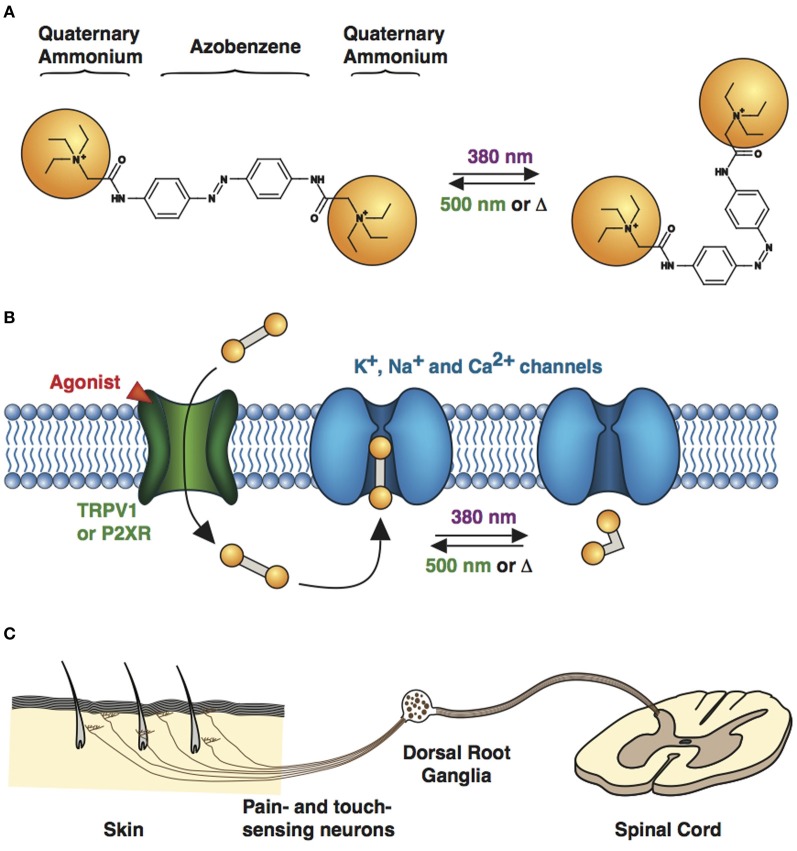
**Cell targeting strategy with QAQ. (A)** Chemical structures of *trans* (left) and *cis* (right) QAQ. **(B)** QAQ is membrane impermeable, but can enter cells through open TRPV1 or P2XR channels. Once in the cystosol, QAQ photosensitizes voltage-gated K^+^, Na^+^, and Ca^2+^ channels. **(C)** Touch- and pain-sensing neurons innervate the skin and project to the spinal cord. Their cell bodies are located in the dorsal root ganglia. QAQ can be targeted specifically to pain-sensing neurons, which contain high densities of TRPV1 channels.

QAQ is a doubly charged (Figure 3A), membrane-impermeant PCL that does not enter most cells when applied extracellularly. However, we found that QAQ was able to enter cells through open TRPV1 and some P2X channels, enabling the targeted photosensitization of cells expressing either of these channels (Mourot et al., 2012). TRPV1 channels are heat-activated channels required for the detection of noxious heat (Caterina et al., 1997), while P2X receptors are ion channels activated by extracellular ATP (Valera et al., 1994). One biophysical property shared by these two ion channel types is their ability to allow large organic cations to pass through the channel pore (Khakh et al., 1999; Virginio et al., 1999; Meyers et al., 2003; Chung et al., 2008; Binshtok et al., 2007). We took advantage of this property to deliver QAQ into specific cells, utilizing these channels as “molecular conduits” (Figure 3B).

TRPV1 channels are highly expressed in pain fibers but scarcely present in the central nervous system (CNS) (Arenkiel et al., 2008; Cavanaugh et al., 2011). P2X receptors are primarily expressed in the peripheral nervous system and in epithelial and endothelial tissue, but are also present to some extent at a few central synapses (Khakh and North, 2006). Because both channels and their respective agonists, capsaicin and ATP, are nearly absent from the CNS, heterologous expression of TRPV1 or P2X channels could be used as an orthogonal way of optically silencing genetically identified neurons with QAQ (Mourot et al., 2012). Miesenböck and colleagues designed a comparable optochemical genetic strategy to remotely photoactivate specific neurons heterologously expressing TRPV1 or P2X channels, using caged-capsaicin or caged-ATP, in living flies (Zemelman et al., 2003; Lima and Miesenbock, 2005; Claridge-Chang et al., 2009). While elegant and powerful, both approaches suffer from the technical complexities associated with the expression of foreign genes and the subsequent delivery of photosensitive chemicals.

However, exogenous expression of TRPV1 or P2X receptor is not always necessary to target QAQ-induced photosensitivity to specific neurons. We took advantage of the intrinsic TRPV1 expression pattern to specifically deliver QAQ to pain-sensing neurons in rodents. Co-application of QAQ and capsaicin resulted in the selective entry of QAQ into pain-sensing neurons (aka nociceptors) both *in vitro* and *in vivo*, without affecting neighboring cells such as touch-sensing neurons, which are devoid of TRPV1 channels (Mourot et al., 2012). Sensory neurons are pseudo-unipolar neurons, with a peripheral process that carries AP to the cell body (located in the dorsal root ganglia), and a central process that carries AP to the post-synaptic neuron in the spinal cord (Figure 3C). The peripheral axons of nociceptors are usually extremely long and mixed together with other sensory neurons. Hence it has been extremely difficult to manipulate the activity of these neurons in a specific manner using current electrophysiological or pharmacological tools. Because QAQ can enter nociceptors at the level of their cell bodies, their synaptic terminals or their sensory nerve endings, our method enables the rapid control of pain signaling with a high degree of precision. This method will undoubtedly prove useful for understanding pain circuitry and pain signaling, in both physiological and pathological conditions.

## Two-component systems: development of designer photoreceptors

The various voltage- and ligand-gated ion channels found in neurons have very distinct biophysical properties as well as different subcellular and cellular distributions, but often share overlapping pharmacology. The limited selectivity of pharmacological agents has hindered the understanding of their physiological roles. Traditional drugs often cannot differentiate between different receptor types or subtypes. Some of them, such as toxins, do show good receptor selectivity, but their temporal action profile cannot mimic the rapid rise and fall in neurotransmitter concentrations in the brain, as they persist in the tissue for an extended period of time and cannot be washed out quickly. In addition, the effect of traditional pharmacological agents cannot be easily restricted to a specific cell type or tissue; for example, there is no method for selectively controlling post- vs. pre-synaptic receptors. The relationship between receptor function and behavior can also be studied via the generation of transgenic organisms, but this strategy lacks temporal specificity and can produce animal phenotypes where undesirable developmental defects and compensatory changes are superimposed on the true effects of receptor removal. The development of spatially and temporally targeted activation/inactivation of specific receptors will help to overcome this problem and produce a better understanding of the function of receptors in adult animals.

A number of chemical genetic strategies have been developed to overcome the limitations of conventional pharmacology. They combine the powers of chemistry and genetic targeting to enable an unparalleled degree of pharmacological specificity (Rogan and Roth, 2011; Wulff and Arenkiel, 2011). One strategy involves designing a combination of chemically modified small molecule ligands and mutant receptors that specifically bind these ligands. The modified ligands are considered “orthogonal” in normal cells, since they should not bind any wild-type receptor, but only their mutant partner. This approach was first demonstrated on a mutant κ opiod receptor, which was engineered to dramatically reduce its affinity for the endogenous ligand dynorphin, but still respond to synthetic small molecule drugs (Coward et al., 1998). This type of receptor activated solely by synthetic ligands was called a RASSL. The receptor-ligand pair strategy was later extended to create other mutant G protein-coupled receptors with lower baseline activity. These second generation designer receptors exclusively activated by designer drugs were named DREADDs (Armbruster et al., 2007). Ligand-gated ion channels, including GABARs (Wulff et al., 2007) and nAChRs (Magnus et al., 2011) were also engineered to be orthogonal to certain ligands. In the case of nAChRs, a variety of pharmacologically selective effector molecules (PSEMs) and pharmacologically selective actuator modules (PSAMs) were generated, with different ionic permeabilities for remotely exciting or silencing targeted neurons. The mutant receptors may retain their original function, in which case the receptor-ligand pair is used to define the biological roles of the receptor (Wulff et al., 2007). Conversely, the receptor may only possess the ability to bind the orthogonal ligand but not the endogenous neurotransmitter, in which case the receptor becomes orthogonal as well and is used to remote control cellular signaling or membrane excitability in genetically targeted cells (Armbruster et al., 2007; Magnus et al., 2011). However, though this chemical genetic approach has produced highly specific receptor-ligand combinations that work well both *in vitro* and *in vivo*, it still suffers from the lack of fine spatiotemporal control of receptor activity, due to the inability to control ligand diffusion into or out of a region of interest or manipulate the dynamics of ligand binding/unbinding. Light is the perfect modality for achieving such precise spatiotemporal control, as it allows for micrometer spatial and millisecond temporal resolution. We thus decided to combine the power of chemical genetics with the precision of optical control in order to develop a series of photoswitchable tethered ligands (PTLs) for the optochemical genetic control of ion channels (Fehrentz et al., 2011).

The PTL strategy involves the genetic engineering of receptors and their conjugation to a chemical photoswitch, allowing light to precisely activate or inhibit that receptor subtype in a given neuronal population. The PTL contains a ligand (agonist or pore blocker/antagonist) on one end of the molecule, a central photoisomerizable azobenzene core, and a cysteine-reactive group on the other end of the molecule. The PTL can react with engineered cysteine point mutations in the ion channel of interest, covalently tethering the ligand to the receptor. In addition to the shape and dipole moment changes, the end-to-end distance of the *trans* and *cis* isomers is also substantially different, allowing light to toggle the ligand in and out of its binding pocket. Photoisomerization of the PTL thus leads to a reversible activation or inhibition of the receptor. Such a strategy allows us to control an individual ion channel subtype in a neuron, i.e., the one containing the cysteine mutation, with high spatiotemporal precision, while leaving native channels unaffected. Importantly, the functionality of the mutant receptor should remain unchanged prior to PTL treatment, but its activity would then be controlled by light after conjugation of the PTL. Because designer channels can be genetically targeted, they belong to the family of optogenetic tools, even though they need a photochemical co-factor for functioning.

**Figure 4:**
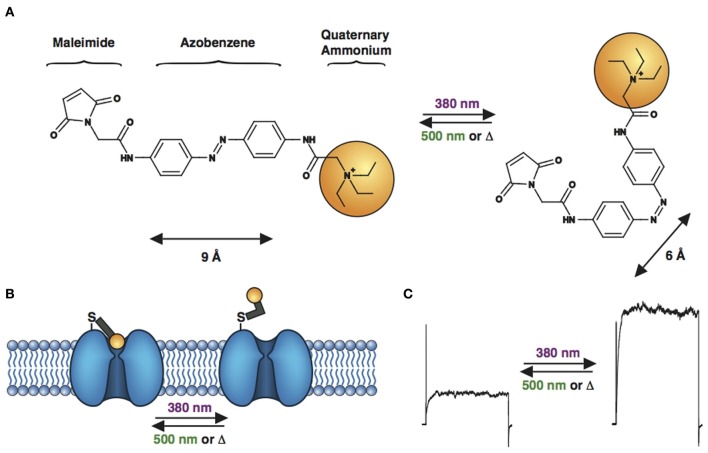
**Engineered potassium channels with a photoswitchable tethered ligand (PTL). (A)** Chemical structures of *trans* (left) and *cis* (right) MAQ (Maleimide-Azobenzene-Quaternary ammonium). The end-to-end distance between the *para* positions of the azobenzene shortens by ~3 Å upon isomerization from *trans* to *cis*. **(B)** Scheme of a genetically-encoded, photoswitchable K^+^ channel. MAQ is covalently attached on an engineered cysteine located on the extracellular surface of a K^+^ channel. In the extended *trans* configuration (dark or 500 nm light), MAQ blocks ion conduction. Photoisomerization to *cis* with 380 nm light shortens MAQ and relieves block. **(C)** Current through a photoswitchable Kv 3.1 channel labeled with MAQ, under both wavelengths of light.

PTLs were first designed for the voltage-gated Shaker K^+^ channel, resulting in synthetic photoisomerizable azobenzene-regulated K^+^ (SPARK) channels (Banghart et al., 2004; Chambers et al., 2006) that could be blocked and unblocked by light. SPARK channels are cysteine-containing mutant Shaker K^+^ channels, which can be conjugated to a photoswitchable ligand, maleimide-azobenzene-quaternary ammonium (MAQ, Figure 4A), containing the pore blocker QA. QAs not only block K^+^ channels at the level of the inner vestibule, but also at an external binding site located just outside the selectivity filter (Hille, 2001). After MAQ conjugation, the QA moiety can reach the ion channel's outer pore only in the *trans* but not the *cis* configuration, which results in conduction block under 500 nm light or in the dark, when the PTL is in the *trans* form (Figures 4B,C). Illumination with 380 nm light converts the PTL to its shorter *cis* form, unblocking the ion channel and allowing for current flow. At low micromolar concentration, MAQ does not cross the cell membrane, ensuring specific optical control of the engineered cysteine mutant channel (Fortin et al., 2011; Sandoz et al., 2012). Expression of SPARK channels in neurons enabled the photocontrol of neuronal activity (Banghart et al., 2004; Chambers et al., 2006). However, because Shaker is a *Drosophila* K^+^ channel, it is orthogonal to mammalian neurons and is therefore unsuited for the study of endogenous K^+^ channel function in the mammalian CNS. We have thus decided to transpose the strategy to mammalian K^+^ channels. Tethering MAQ onto engineered cysteine mutants of K_v_1.3, K_v_3.1, K_v_7.2, Ca^2+^-activated SK2 and TREK1 conferred photosensitivity on these K^+^ channels (Fortin et al., 2011; Sandoz et al., 2012). These novel light-sensitive channels are highly interesting targets for optical control. For example, K_v_7.2 is a potassium channel underlying the M-current and has also been implicated in epilepsy (Brown and Passmore, 2009), while the calcium-activated SK2 channel modulates excitatory post-synaptic potentials and contributes to long-term potentiation in the hippocampus (Adelman et al., 2011). TREK1 channels are polymodal channels, being sensitive to temperature, pressure, pH and intracellular signaling (Alloui et al., 2006). Specific photomodulation of TREK1 revealed that they are also activated by GABA_B_ signaling in the hippocampus (Sandoz et al., 2012).

K^+^ channels are not the only family of transmembrane proteins that can be targeted with the PTL strategy. We have recently developed a series of light-regulated neuronal nAChRs (LinAChRs) which can be conjugated with photoswitchable agonists or antagonists, allowing for powerful bidirectional control of receptor activity (Tochitsky et al., 2012). Given the wide variety of nAChR subtypes present in the brain and the lack of specific pharmacology for them, LinAChRs should prove highly useful in studying the function of nAChRs in the nervous system. Furthermore, controlling specific nAChRs in the brain may help shed light on their role in nicotine addiction and various neurodegenerative and neuropsychiatric disorders. Another major class of channels targeted with PTLs is the glutamate receptor (GluR) family. A series of ionotropic glutamate receptors belonging to the kainate receptor family (iGluR6) could be activated by light after conjugation of a photoswitchable agonist to an engineered mutant cysteine residue (Volgraf et al., 2005; Gorostiza et al., 2007; Numano et al., 2009). The resulting light-activated glutamate receptors (LiGluRs and HyLighter) enabled the remote control of neuronal electrical activity both *in vitro* (Szobota et al., 2007) and *in vivo* in zebrafish larvae (Janovjak et al., 2010; Wyart et al., 2010) and in mice (Caporale et al., 2011).

The success and relative ease of generating novel light-sensitive ion channels demonstrate the power of the PTL approach, as almost any channel or receptor can, in principle, be rendered light-sensitive by combining existing pharmacological and structural information. Synthetic organic chemistry can generate photosensitive variants of existing receptor ligands. Molecular modeling can then identify a region of interest where cysteine scanning mutagenesis would determine the optimal position for PTL attachment. Once a particular channel has been photosensitized, structurally similar relatives can be photosensitized as well, often simply by transposing the cysteine mutation from one subtype to another (Fortin et al., 2011; Tochitsky et al., 2012). A PTL-conjugated receptor can be controlled specifically, with either an agonist or antagonist/pore blocker, with a spatial and temporal precision simply impossible with traditional diffusible ligands. The combination of rational design, specificity and precision makes the PTL approach a very powerful tool for identifying the roles of particular ion channel subtypes in complex neural networks, both *in vitro* and *in vivo*.

So far, the designer receptor strategy has mainly been used *in vitro*, where gene delivery and conjugation of the PTL onto the engineered cysteine mutation are relatively staightforward. *In vivo*, its use has been restricted to zebrafish larvae where the PTL can be added to the water where fish swim and absorbed through the skin (Janovjak et al., 2010; Wyart et al., 2010) and to the mouse retina where the PTL can be easily injected into the vitreous cavity of the eye (Caporale et al., 2011). Traditional optogenetic tools (e.g., channelrhodopsin) have an advantage over designer receptors in this regard, since the photoswitch retinal is naturally present in cells and does not have to be introduced exogenously. Nonetheless, designer receptors can be used to answer questions that would be hard to address with channelrhodopsin. For example, engineered receptors can reveal a causal relationship between receptor activation in a given cell type and the activity of a neuronal circuit or the induction of a certain behavior. Light-sensitive designer receptors can also be used to investigate the function of receptors with different subunit compositions, or the function of a specific receptor expressed in different cell types. However, before these new optical tools can be generally used in mammals *in vivo*, a certain number of challenges must be overcome:

*Gene delivery:* photosensitive ion channels can be overexpressed in neurons and manipulated with light in order to study their function, but it is preferable to maintain the endogenous level of gene expression in order to ensure that the results are physiologically relevant. Such a system can be created via the generation of a *knock-in* animal, where the endogenous channel is replaced by the cysteine mutant, although this approach is laborious and costly. Alternatively, the cysteine mutant subunit can be expressed in a system where it would need to coassemble with an endogenous wild-type subunit for trafficking to the plasma membrane. This photoswitchable conditional subunit strategy was successfully utilized *in vitro* for the photosensitive TREK1 two pore K^+^ channel (Sandoz et al., 2012) and should be feasible *in vivo* for other heteromeric ion channels such as brain nAChRs. Expression of the cysteine-containing subunit can in principle be done in the wild-type background, although the use of a *knock-out* animal is preferred to maximize the degree of photocontrol. *In vivo* gene delivery can be done using lenti- or adeno-associated (AAV) viral vectors, or the *in utero* electroporation technique (Fenno et al., 2011).
*Cell-type specific expression:* one method of manipulating genetically defined neuronal subtypes involves the expression of the gene of interest under the control of cell-type specific promoters. Another possibility is to use Cre recombinase-inducible expression systems in conjunction with transgenic animals expressing Cre in specific neuronal populations. Cre recombinase will catalyze the recombination between two *loxP* sites, resulting in the reversal of the gene's orientation and allowing for the gene's mRNA to be transcribed. High levels of cell type specificity can usually be achieved with this technique (Fenno et al., 2011).
*Light delivery:* since the advent of optogenetics, different hardware setups have been used to deliver light *in vitro* and *in vivo* (Fenno et al., 2011). Lasers coupled to optic fibers are most commonly used to deliver light into the brain. The fiber can be placed either in the same brain area as the virus injection or in projection targets.
*PTL delivery:* one of the biggest challenges of our PTL approach is the delivery of the photoswitch. Because of maleimide's reactivity, delivery has to be done locally into the brain, using a guide cannula for example. The cannula can be chronically implanted into the animal, allowing for the delivery of both the chemical photoswitch and the optical stimulation fiber to the region of interest. Since it covalently tethers to its receptor, the PTL can be applied hours before an experiment, ensuring that the excess non-conjugated PTL will diffuse or be metabolized. Another important factor to consider is the efficiency of conjugation, which depends on the oxidation state of the cysteines. Reducing agents such as DTT or TCEP have been used *in vitro* to reduce cysteines and increase labeling; it is not clear whether these agents will also be required for *in vivo* use.

## Biomedical applications

Gene therapy with optogenetic tools has been proposed as a strategy for restoring vision (Busskamp and Roska, 2011) as well as for treating Parkinson's disease or epilepsy through deep brain optical stimulation (Gradinaru et al., 2009). Although current gene therapy trials for CNS diseases have shown good safety, it is not clear how the human immune system will tolerate the high levels of microbial opsin expression required for photosensitizing neurons. In contrast, due to its non-genetic nature, the PCL approach for photosensitizing neurons should provide a readily reversible, safe therapeutic strategy. Here we describe the potential use of PCLs for the treatment of blindness and management of chronic pain.

### Vision

**Figure 5:**
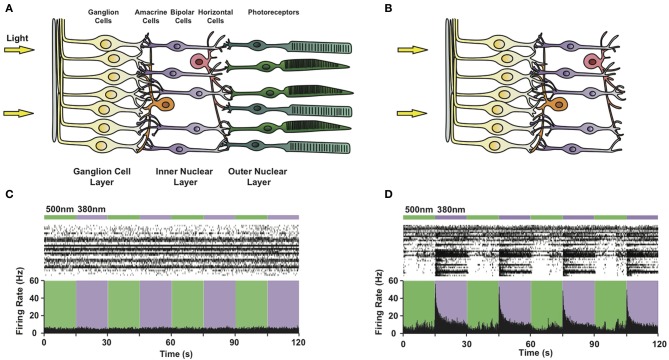
**Photosensitizing blind retinas with PCLs.** Circuit diagram of **(A)** a healthy retina and **(B)** a retina lacking rod and cone photoreceptors due to advanced degeneration. Note that retinal ganglion, bipolar and amacrine cells survive photoreceptor death. **(C,D)** Multi-electrode array recordings from a flat-mounted *rd1* mouse retina **(C)** before and **(D)** after treatment with 300 μ M AAQ. Top: Color bars representing illumination with 380 nm (violet) or 500 nm (green) light. Middle: Raster plot of RGC activity. Bottom: Average RGC firing rate (bin size = 100 ms).

Given that the use of PCLs for clinical applications is limited by the ability to deliver light to a particular tissue or organ, the visual system was selected as an ideal clinical target due to the inherent ease of light delivery to the eye. Restoration of neural light sensitivity is needed to treat several degenerative retinal disorders, such as retinitis pigmentosa and age-related macular degeneration, which are characterized by the progressive loss of rod and cone photoreceptors, eventually leading to complete blindness (Jones et al., 2012). In such end-stage blinding diseases, the photoreceptors are absent but other types of neurons in the retina, including retinal ganglion cells (RGCs), bipolar and amacrine cells remain intact (Figures 5A,B). We decided to test whether treatment of the blind retina with AAQ would confer light sensitivity onto these remaining neurons and whether this engineered light response could drive visually guided behavior *in vivo*.

To evaluate the effect of the PCL, we selected the *rd1*/*rd1* mouse model of end-stage retinitis pigmentosa, in which virtually all photoreceptors die by 3 months after birth (Sancho-Pelluz et al., 2008). Prior to AAQ treatment, no light responses were observed on the multi-electrode array (MEA) (Figure 5C), which records the activity of dozens of RGCs *in vitro*. After treatment with AAQ, we observed a dramatic increase in RGC activity upon stimulation with 380 nm light, which could be turned off by subsequent 500 nm illumination (Figure 5D) (Polosukhina et al., 2012). This pattern of AAQ-mediated light responses was opposite to what we had previously observed in cultured neurons, where 380 nm light usually decreases neuronal excitability (Figure 2A). By applying pharmacological synaptic blockers, we determined that AAQ photosensitized all remaining retinal cell types, including the inhibitory amacrine cells. After blockade of inhibitory GABAergic and glycinergic inputs, the RGCs increased their activity in 500 nm light, consistent with previous findings from cultured neurons (Polosukhina et al., 2012). The photo-control of inhibitory amacrine synaptic drive thus appears to be the major contributor to light-dependent RGC activity after AAQ treatment.

Amazingly, intraocular injection of AAQ could also restore visually guided behaviors in *rd1* mice *in vivo*. After the injection, previously blind mice displayed light aversive locomotory behavior in a turning assay. Additionally, these mice regained a light-driven pupillary light reflex. Finally, AAQ-injected *rd*1/*rd*1 mice displayed light-dependent changes in locomotory activity in an open-field assay. The ability of AAQ to restore light perception to blind mice *in vivo* makes the PCL approach a promising potential therapy for end-stage retinitis pigmentosa and age-related macular degeneration. Novel K_v_ PCLs such as DENAQ and PHENAQ (Mourot et al., 2011) have a red-shifted absorption spectrum and faster thermal relaxation compared to AAQ. These molecules may be better clinical candidates due to their ability to respond to visible rather than UV light and be controlled with a single wavelength of light. Further chemical synthesis and screening of our existing PCL library should allow us to develop compounds with optimal properties for vision restoration—ones sensitive to dim visible light, safe for intraocular use and producing long-lasting photosensitivity *in vivo* after a single injection.

### Chronic pain

Local anesthetics such as lidocaine are potent silencers of neuronal activity and extremely useful clinical drugs for managing acute pain. However, these drugs are not well suited to the treatment of persistent, chronic pain because (1) they lack specificity for motor vs. sensory neurons and also for different sensory modalities (e.g., touch sensation) (2) block duration or intensity cannot be regulated (Roberson et al., 2011). Because QAQ is selective for pain-sensing neurons, it may block nociception without affecting motor axons or other sensations. Moreover, because QAQ blockade can be precisely modulated by changing light wavelength or intensity, it may be possible to photo-titrate the analgesic effect at will.

QAQ could be administered in combination with capsaicin to selectively block TRPV1-containing nociceptors. A similar strategy has been proposed for the membrane-impermeant lidocaine derivative QX-314, which produces long-lasting selective analgesia (Binshtok et al., 2007; Kim et al., 2010). However, because capsaicin activates TRPV1 channels and produces pain, it may be desirable to target nociceptors by other means (Roberson et al., 2011). In pathological pain conditions, nociceptors become hyperactive. Our experiments with QAQ suggest that the basal activity of TRPV1 channels increases in electrically hyperactive neurons (Mourot et al., 2012). If this is the case, capsaicin may not be needed, and QAQ may be self-targeted to the most active nociceptors, i.e., the ones that need to be silenced the most. This finding may explain why QX-314 alone, which is normally unable to cross neuronal membranes, is effective for recovery after spinal cord injury (Agrawal and Fehlings, 1997) and in animal models of neuropathic pain (Omana-Zapata et al., 1997).

Photoregulation of pain sensation *in vivo* requires light delivery to the targeted neuronal tissue. Because the terminal endings of nociceptive neurons are located just below the skin, an external light source could be used after topical application of QAQ. The cornea, which is fully transparent and highly-enriched in sensory fibers, is the ideal peripheral target, as evidenced by the potent optical regulation of touch-evoked responses seen in living rats (Mourot et al., 2012). Red-shifted derivatives of QAQ (Mourot et al., 2011) may be more appropriate for the optical regulation of pain deeper in the skin or in internal structures such as the spinal cord, in combination with implanted fiber-optic systems.

## Conclusion

We have developed multiple one-component PCL and two-component PTL systems for photosensitizing ion channels naturally present in neurons. Both of these strategies work well *in vitro* and *in vivo*. Chemical PCLs render many types of neurons sensitive to light within minutes without requiring exogenous gene expression. They are photosensitive pharmacological agents, the activity of which can be rapidly turned on or off with light. As such, they provide a unique means to quickly and precisely reverse the activity of drugs in cases where simple perfusion would be slow, tricky or impossible (e.g., the patch pipette). They are also promising clinical candidates for treating blindness and pathological pain. In contrast, PTLs are highly sophisticated molecular tools that enable the optical control of a defined ion channel in a targeted cell population. This ability to specifically control a given receptor in a defined neuronal population and monitor changes in behavior due to its activation or inhibition should provide an unprecedented ability to uncover the functional connectivity of the nervous system. Our studies will thus be crucial to the design of novel, targeted drugs for treating neurological and neuropsychiatric disorders.

### Conflict of interest statement

The authors declare that the research was conducted in the absence of any commercial or financial relationships that could be construed as a potential conflict of interest.

## Abbreviations

AAQ: acrylamide-azobenzene-quaternary ammonium
AIS: Axon Initial Segment
BzAQ: Benzylamide-azobenzene-quaternary ammonium
Ca_v_: voltage-gated calcium channel
DENAQ: diethylamine-azobenzene-quaternary ammonium
DREADDs: designer receptors exclusively activated by designer drugs
GABARs: gamma-amino butyric acid receptors
HCN: hyperpolarization-activated cyclic nucleotide-gated
iGluR: ionotropic glutamate receptor
K_ir_: inward-rectifier potassium channel
K_v_: voltage-gated potassium channel
MAQ: maleimide-azobenzene-quaternary ammonium
nAChR: nicotinic acetylcholine receptor
Na_v_: voltage-gated sodium channel
PCL: photochromic ligand
PhENAQ: phenylethylamine-azobenzene-quaternary ammonium
PrAQ: propyl-azobenzene-quaternary ammonium
PSAM: pharmacologically selective actuator modules
PSEM: pharmacologically selective effector molecules
PTL: photoswitchable tethered ligand
QA: quaternary ammonium
QAQ: quaternary ammonium-azobenzene-quaternary ammonium
RASSLs: receptors activated solely by synthetic ligands
RGC: retinal ganglion cell.
